# The efficacy of islet autoantibody screening with or without genetic pre-screening strategies for the identification of presymptomatic type 1 diabetes

**DOI:** 10.1007/s00125-025-06408-4

**Published:** 2025-03-19

**Authors:** Ezio Bonifacio, Raquel Coelho, Domenik A. Ewald, Gita Gemulla, Michael Hubmann, Przemyslawa Jarosz-Chobot, Mirjam Kohls, Olga Kordonouri, Vito Lampasona, Parth Narendran, Flemming Pociot, Zdenek Šumník, Agnieszka Szypowska, Jose Zapardiel-Gonzalo, Anette-Gabriele Ziegler

**Affiliations:** 1 Center for Regenerative Therapies Dresden, Faculty of Medicine, TU Dresden, Dresden, Germany; 2 Pediatric Department, APDP-Diabetes Portugal, Education and Research Center (APDP-ERC), Lisboa, Portugal; 3 Practice for Paediatric Medicine, Regensburg, Germany; 4 Department of Paediatrics, University Hospital Carl Gustav Carus, Dresden, Germany; 5 Practice for Paediatric Medicine, Zirndorf, Germany; 6 Department of Children’s Diabetology and Lifestyle Medicine, Medical University of Silesia, Katowice, Poland; 7 Institute of Diabetes Research, Helmholtz Munich, Munich, Germany; 8 Department of Pediatrics, Diabetology, Endocrinology, and Clinical Research, Kinder- und Jugendkrankenhaus AUF DER BULT, Hannover, Germany; 9 Diabetes Research Institute, IRCCS Ospedale San Raffaele, Milan, Italy; 10 Department of Immunology and Immunotherapy, University of Birmingham, Birmingham, UK; 11 Clinical and Translational Research, Steno Diabetes Center Copenhagen, Herlev, Denmark; 12 Department of Pediatrics, 2nd Faculty of Medicine, Charles University in Prague and Motol University Hospital, Prague, Czechia; 13 Department of Paediatric Diabetology and Paediatrics, Medical University of Warsaw, Warsaw, Poland

## Abstract

Early detection of type 1 diabetes, in its presymptomatic stage, offers significant clinical advantages, including treatment that can delay disease onset. Current screening focuses on identifying islet autoantibody positivity, with proposed optimal testing at ages 2, 6 and 10 years potentially achieving up to 80% sensitivity. However, challenges arise from participation rates and costs associated with multiple screenings. Genetic pre-screening has been suggested as a complementary strategy to target high-risk individuals prior to autoantibody testing, but its real-world benefits remain uncertain. Broad genetic selection strategies, based on family history, HLA typing or polygenic risk scores, can identify subsets of the population at elevated risk. However, these approaches face issues like low recall rates, socioeconomic biases and limited applicability across diverse ancestries. Additionally, the cost-effectiveness and infrastructure requirements of integrating genetic testing into routine healthcare remain significant hurdles. The combined use of genetic and autoantibody testing could improve predictive value, especially with innovations like point-of-care genetic testing. Yet, the ultimate success of any screening programme depends less on specific strategies and more on maximising public and healthcare-provider engagement, ensuring high participation, and addressing socioeconomic and demographic disparities. Digital-health infrastructure may play a crucial role in improving recall rates and maintaining follow-up adherence. In conclusion, while repeated islet autoantibody screening remains the most effective standalone approach, conducting genetic screening prior to islet autoantibody testing may be practical in certain contexts, provided that sufficient resources and equitable strategies are employed. Public engagement and robust infrastructure are essential to realising the full potential of early type 1 diabetes detection programmes.

**Graphical Abstract:**

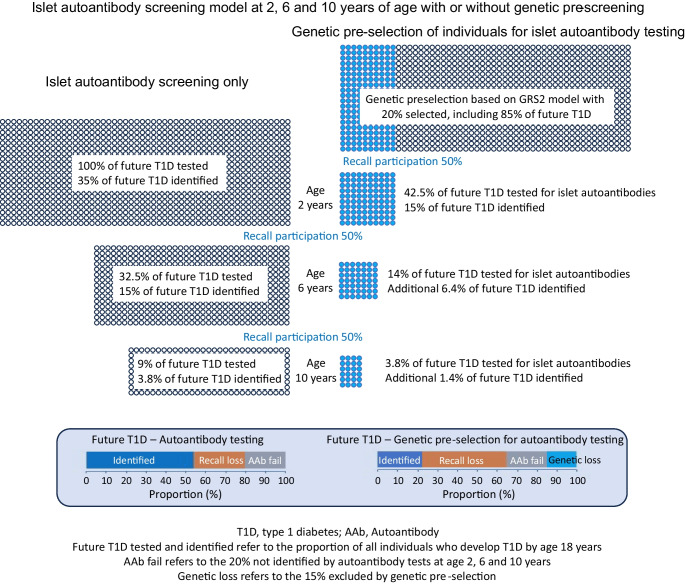

**Supplementary Information:**

The online version contains peer-reviewed but unedited supplementary material available at 10.1007/s00125-025-06408-4.

## Background

Screening for presymptomatic early-stage type 1 diabetes has garnered significant attention due to the recent availability of teplizumab, which delays the clinical onset of the disease [1] and the demonstrated clinical benefits associated with an early diagnosis [2, 3]. Screening aims to identify individuals who are persistently positive for two or more islet autoantibodies, which defines the presymptomatic stage of type 1 diabetes [4]. Screening for early-stage type 1 diabetes via testing in children from the general population for islet autoantibodies has now been adopted in several partner regions within the European action for the Diagnosis of Early Non-clinical Type 1 diabetes for disease Interception (EDENT1FI) consortium [5–8].

## When to screen for islet autoantibodies

It has been proposed that the optimal times for islet autoantibody screening are at 3 years of age if screening is performed once, or at 2 and 6 years of age if two rounds of islet autoantibody screening are performed [9, 10]. Screening once at 3 years of age, during the presymptomatic stage of type 1 diabetes, identifies around 35% of individuals who will develop clinical type 1 diabetes by the time they are 18 years old (sensitivity), while screening at ages 2 and 6 years increases the sensitivity of screening to approximately 65%. A further screen at 10 years of age captures the majority of individuals who will develop type 1 diabetes by the time they are 18 years of age [11]; consequently, the sensitivity of screening would potentially increase to around 80% if it is performed at 2, 6 and 10 years of age (Table 1 and Electronic supplementary material [ESM] Table 1 [data in ESM Table 1 are from [9–11]]). Second and third screening rounds will incur substantial additional costs and sensitivity will be affected by low recall rates.

**Table 1:** Estimated performance of islet autoantibody testing to identify type 1 diabetes in its presymptomatic early stage

Antibody testing strategy	Early-stage type 1 diabetes^a^	Sensitivity^b^	Total tests per 100,000 people
Single screen (at age 3 years)	0.2%	35%	100,000
Two-age screen (at 2 and 6 years)	0.4%	65%	199,800
Three-age screen (at 2, 6 and 10 years)	0.5%	80%	299,400

^a^Based on updated numbers from screening in the Fr1da study [5, 24]

^b^Defined as proportion of youth who develop type 1 diabetes by 18 years of age. Sensitivities are estimates based on screening performed in the TEDDY study and the Type 1 Diabetes Intelligence cohort [9–11]

## Genetic pre-screening

Incorporating an a priori genetic screen into islet autoantibody screening programmes to select those with increased genetic risk has been suggested as a way to reduce the number and, consequently, cost of islet autoantibody testing, [12]. However, the benefit of such an approach in terms of increasing the proportion of cases detected in the presymptomatic phase or reducing costs is uncertain. Here, we examine the empirically estimated efficacy of islet autoantibody screening performed with and without genetic selection in youth and discuss practical aspects of both approaches.

### Genetic pre-screening strategies

Genetic testing prior to islet autoantibody screening has value if it offers reasonable discrimination. For type 1 diabetes, selection can be achieved through use of family history, HLA typing and polygenic risk scores in children and adolescents [13, 14]. To capture the majority of those who develop clinical diabetes, it will be necessary to adopt a broad genetic selection approach. For example, this could include all youth with a first-degree relative with type 1 diabetes or with *HLA-DR3* or *HLA-DR4-DQ8* haplotypes (herein referred to as genetic selection ‘strategy A’), which together would cover approximately 90% of type 1 diabetes cases (Table 2 and ESM Table 1). However, depending on the population frequency of risk HLA haplotypes in the region, this would select around one-third of youth to be counselled and, subsequently, recalled for islet autoantibody testing on multiple occasions. Notably, the overall risk in those selected for islet autoantibody testing would be relatively low (1.2% or threefold of the unscreened population). Assuming 100% recruitment and 100% recall at each of three screening stages (at 2, 6 and 10 years of age), such a strategy will require almost 200,000 total genetic plus islet autoantibody tests per 100,000 youth and could achieve 72% sensitivity. Selection using more sophisticated genetic risk scores (e.g. GRS2 >80th centile [12]) that identify 20% of the population for follow-up islet autoantibody screening, covering approximately 85% of type 1 diabetes cases (overall risk in individuals selected: 1.6%), as suggested by genetic selection ‘strategy B’ [11] (Table 2 and ESM Table 1), would require close to 160,000 total tests and could achieve 68% sensitivity. Any further stringency on the genetic selection to increase the risk threshold for those tested, for example genetic selection ‘strategy C’ which identifies individuals with a first-degree relative with type 1 diabetes, or *HLA-DR3/DR4-DQ8* or *HLA-DR4-DQ8/DR4-DQ8* genotypes for follow-up islet autoantibody screening, would result in further loss of sensitivity (Table 2 & ESM Table 1).

**Table 2:** Estimated performance of genetic plus islet autoantibody testing to identify type 1 diabetes in its presymptomatic early stage

Strategy	Proportion with follow-up islet AAb tests^a^	Risk^b^	Sensitivity^c^	Genetic plus islet AAb tests per 100,000 people
Genetic selection followed by islet AAb testing for those at genetic high risk of T1D (at age 2, 6 and 10 years)				
Strategy A: genetic selection of individuals with first-degree relative with T1D or *HLA-DR3* or *HLA-DR4-DQ8*	33% (90% of T1D)	1.2% (3-fold)	72%	198,555
Strategy B: genetic selection of individuals with GRS2 >80^th^ centile [12]	20% (85% of T1D)	1.6% (4-fold)	68%	159,602
Strategy C: genetic selection of individuals with first-degree relative with T1D or *HLA*-*DR3/DR4-DQ8* or *HLA-DR4-DQ8/DR4-DQ8*	3% (35% of T1D)	4.8% (12-fold)	28%	108,848
Combined genetic and islet AAb testing				
Strategy D: genetic and islet AAb testing at 2 years of age in whole population, with follow-up islet AAb screening at 6 and 10 years of age in individuals with a first-degree relative with T1D, or *HLA-DR3/DR4-DQ8* or *HLA-DR4-DQ8/DR4-DQ8*	3% (35% of T1D)	4.8% (12-fold)	51%	205,671

^a^Data derived from newborn screening in the GPPAD-02 study [15]

^b^Based on a population risk for type 1 diabetes by the age 18 years of 0.4%. Fold values relative to the background (non-screened) population

^c^Defined as the proportion of all individuals who will develop clinical type 1 diabetes by 18 years of age, who are identified by the screening strategy. Sensitivities are estimates based on screening performed in the TEDDY study and the Type 1 Diabetes Intelligence cohort [9–11], adjusted for the proportion of type 1 diabetes identified by genetic screening

AAb, autoantibody; T1D, type 1 diabetes

### Limitations of genetic pre-screening

**Fig. 1:**
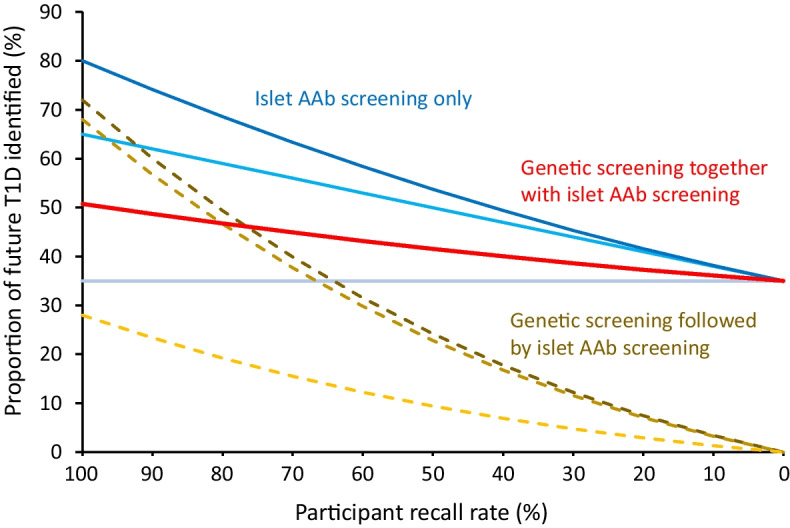
Sensitivity of identifying youth who will develop type 1 diabetes (T1D) by 18 years of age during the presymptomatic early stage of disease, according to recall participation rate. Strategies shown include: (1) islet autoantibody (AAb) screening only (single screen, grey/blue solid line; two-age screen, light-blue solid line; three-age screen, dark-blue solid line); (2) genetic pre-screening with subsequent AAb screening at 2, 6 and 10 years of age in those identified at high genetic risk of T1D (dark-brown dashed line, strategy A [identifying youth with a first-degree relative with T1D or with *HLA-DR3* or *HLA-DR4-DQ8* haplotypes]; light-brown dashed line, strategy B [identifying youth with GRS2 >80th centile [12]]; yellow dashed line, strategy C [identifying individuals with a first-degree relative with T1D or *DR3/DR4-DQ8* or *HLA-DR4-DQ8/DR4-DQ8* genotypes]); and (3) combined genetic and islet AAb testing, followed by islet AAb testing in those with high genetic risk for T1D (i.e. individuals with a first-degree relative with T1D or *HLA-DR3/DR4-DQ8* or *HLA-DR4-DQ8/DR4-DQ8* genotypes; red solid line). The participation rate is assumed to be 100% for the first test (islet AAb or genetic); participant recall rates are defined on the *x*-axis for each subsequent test in the strategy

**Fig. 2:**
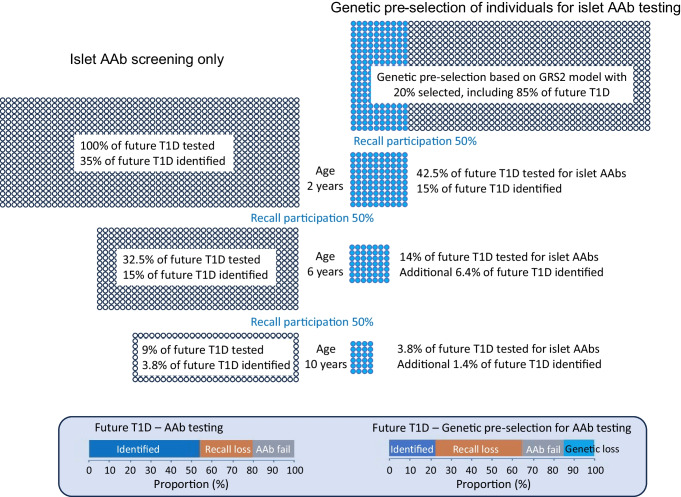
Islet autoantibody (AAb) screening with or without genetic pre-selection. Models are presented for islet AAb screening at age 2, 6 and 10 years without genetic pre-selection, and with genetic pre-selection using the GRS2 at a threshold that selects 20% of children for subsequent islet AAb testing. The models are based on 100% participation at the first screening (of islet AAbs or for genetic pre-selection), and 50% participation loss at each subsequent screening step. The blue filled circles represent those selected by genetic pre-selection. The proportion of future tested refers to the estimated proportion of those who develop type 1 diabetes (T1D) by 18 years of age at each step. The proportion of future T1D identified refers to the estimated proportion of those who develop T1D by 18 years of age that are identified by the screen at each step. A summary of those identified, lost because they did not return for testing (recall loss), and lost because the islet AAb screen failed to identify them (AAb fail) or because they were excluded by the genetic pre-selection threshold (genetic loss) is also shown

While genetic pre-screening offers some reduction in the number of total tests vs islet autoantibody testing alone, a likely major pitfall is that it necessitates recall for islet autoantibody testing, thereby introducing an additional layer of participation loss. Genetic testing has been implemented for enrolment into research studies, such as TEDDY, and clinical trials [15], but enrolment is around 50% or less of those eligible [16]. Genetic risk for type 1 diabetes is not a diagnosis of type 1 diabetes or presymptomatic type 1 diabetes, and the perception of elevated risk by families of children identified as having high genetic risk varies considerably [17]. Therefore, genetic selection strategies are likely to lead to a low return for islet autoantibody testing, as observed in a study from the USA where the participant return rate was less than 10%, although this very low return rate was likely influenced by the coronavirus disease-2019 (COVID-19) pandemic [18]. A low participant return rate is also likely to result in a biased recall population, with a large excess of children who have a family history of type 1 diabetes, and with unequal socioeconomic representation of the general population, particularly if the communicated risk is relatively low, as is the case for strategy A and strategy B. The effect of participant recall loss on the sensitivity of screening is profound for strategies that require genetic selection prior to diagnosis (Fig. 1; ESM Table 1). As an example (Fig. 2), an optimistic strategy B scenario in which 100% of individuals undergo genetic testing at birth as part of a national programme, with 50% recall at each of the three islet autoantibody testing stages (at 2 years, 6 years and 10 years of age), would end up testing for islet autoantibodies in a limited proportion of individuals who go on to develop type 1 diabetes (42.5% at age 2 years, 14% at age 6 years and 3.8% at age 10 years). Using this strategy, 15%, 6.4% and 1.4% of individuals who will develop type 1 diabetes by 18 years of age would be identified at each screening stage, respectively. This participant return rate reduces sensitivity of the strategy to 22.8%, which is less than the sensitivity of a single autoantibody screen. In comparison, for an islet autoantibody screening programme without genetic pre-selection, the proportions of individuals tested who will go on to develop type 1 diabetes would be 100%, 32.5% and 9% when screened at age 2, 6, and 10 years, respectively, identifying 35%, 15% and 3.8% of future type 1 diabetes cases (total sensitivity of 53.8%). One can appreciate how the additional layer of participant recall loss between genetic testing and the first antibody test nullifies any potential benefits of prior genetic testing for subsequent selection strategies for islet autoantibody screening.

The above estimates suggest, therefore, that prior genetic testing is unlikely to offer benefit over autoantibody testing alone in a potential real-world setting, where participant recall loss is likely to be high. Higher participant recall rates may be achieved if genetic test results were automatically linked to digital-health records with alerts to paediatric healthcare providers when children attend clinics or practices. Such infrastructure is likely to be costly. Although actual costs will depend on the extent of genetic markers used, current costs of genetic screening are likely to exceed the costs of islet autoantibody testing, which, in one study, was estimated to be less than €10 (£8) for a single age screen [19]. Cost effectiveness would be improved if broad neonatal genetic testing was introduced into populations [20] and this information could be accessed for early-stage type 1 diabetes screening. Alternatively, increasing acceptance by children and their families and healthcare providers involved in screening and decreasing the cost of a genetic plus islet autoantibody screening strategy could be conceivable with rapid point-of-care genetic tests for specific HLA alleles that are associated with susceptibility to type 1 diabetes, such as *DR3*, *DR4* and *DQ8* [13], as well as some protective HLA alleles. If such point-of-care tests became available and could be performed, for example, using mouth swabs obtained during paediatric visits, they would allow for identification of individuals that require blood collection during the same visit, which will be the minority of the population. This could potentially become a practical solution for reducing recall loss in strategies that conduct islet autoantibody screens after genetic testing. Another option could be to combine genetic and islet autoantibody testing in all children at an early age (e.g. at 2 years of age), with recall only of those with an elevated genetic risk, such as those who have a first-degree relative with type 1 diabetes or with an *HLA-DR3/DR4-DQ8* or *HLA-DR4-DQ8/DR4-DQ8* genotype (genetic selection ‘strategy D’; Table 2 and ESM Table 1). Such a strategy has reasonable sensitivity and may have greater levels of engagement for recall autoantibody testing if the families of children that have been screened are alerted to the substantially higher genetic risk of type 1 diabetes as compared with the background population. A similar strategy to strategy D combined with the GRS2 to define genetic risk has been proposed using data from the TEDDY study [15] but this is yet to be implemented in a research or real-world setting.

## Further considerations

Regardless of when genetic testing is performed, further consideration needs to be given to providing appropriate counselling in those identified as having increased genetic risk of type 1 diabetes. In addition, it is important to consider the anxiety or discrimination that may arise from receiving a genetic risk alert [21], particularly when a sizeable portion of the population may receive this alert (e.g. with genetic strategy A or strategy B). Counselling will incur substantial cost, and a portion of those who have been informed that they have increased genetic risk for type 1 diabetes are likely to refuse counselling. Importantly, it should be considered that much of the evidence and estimates for genetic selection of individuals at risk of type 1 diabetes is based on populations of European descent, which may not be applicable to other populations and, therefore, may be potentially discriminatory. Genetic selection for islet autoantibody screening should, therefore, be tailored to ancestry [22]. Furthermore, the genetic risk estimates used today have been defined using data from individuals who were diagnosed with type 1 diabetes several decades ago and it is likely that the changing type 1 diabetes incidence rates in many countries have resulted in changes to the distribution of type 1 diabetes susceptibility gene alleles, as previously reported [23].

We have not discussed the specificity or positive predictive value of the screening approaches presented here, largely because there is insufficient follow-up data to make reasonable conclusions for children identified as having early-stage type 1 diabetes using general population islet autoantibody screening. Notably, genetic susceptibility was not a significant factor in early progression to clinical diabetes in children identified as having early-stage type 1 diabetes using general population islet autoantibody screening [24]. Nevertheless, it is expected that increasing the prior probability of disease through genetic testing will increase the positive predictive value of a diagnosis of early-stage type 1 diabetes diagnosis for subsequent clinical diabetes. However, a similar improvement in predictive value can be achieved using posterior genetic testing in those who have been identified as multiple-islet-autoantibody positive via screening. Finally, a significant proportion of individuals are diagnosed with type 1 diabetes in adulthood but programmes for screening adults are lacking.

## Conclusions

In summary, we estimate that a screening approach that uses repeated islet autoantibody testing results in the greatest sensitivity for identifying youth who are in a presymptomatic stage of type 1 diabetes. Pre-selection of children who are genetically at risk of type 1 diabetes for islet autoantibody testing is possible, and may be practical in regions where regular paediatric visits are seldom or inconsistently performed, and if genetic screening becomes part of general healthcare. However, additional resources and the infrastructure for genetic tests, notification of risk and counselling, as well as more genetic data in populations of non-European descent, are required. In addition, there will be some loss in sensitivity of islet autoantibody screening approaches if genetic pre-screening is used. For all genetic pre-screening strategies discussed here, high rates of participation and recall for subsequent islet autoantibody testing is of utmost importance to achieve high sensitivity. This is challenging and may require digitalised health records and tracking solutions. Ultimately, the ability to identify a large portion of type 1 diabetes cases during the presymptomatic phase is likely to be less dependent on the screening strategy used, but more reliant on maximising engagement with the public, increasing participation in screening, and with health providers and authorities, to find the most effective ways to reach unbiased and high rates of recall testing.

## Supplementary Information

Below is the link to the electronic supplementary material.

ESM Table 1 (XLSX 23 KB)
